# Supplementary material for: A whole-animal phenotypic drug screen identifies suppressors of atherogenic lipoproteins
Source: eLife. 2026 Aug 27;14:RP105314. doi: 10.7554/eLife.105314 (PMC13521566; doi:10.7554/eLife.105314)
Supplement: Supplementary file 2 [file elife-105314-code1.zip › JHDL_primary_result/3-METHYLCHOLANTHRENE/3-METHYLCHOLANTHRENE_JHDL_7_026_016_2019_06_07_12_30.pdf]

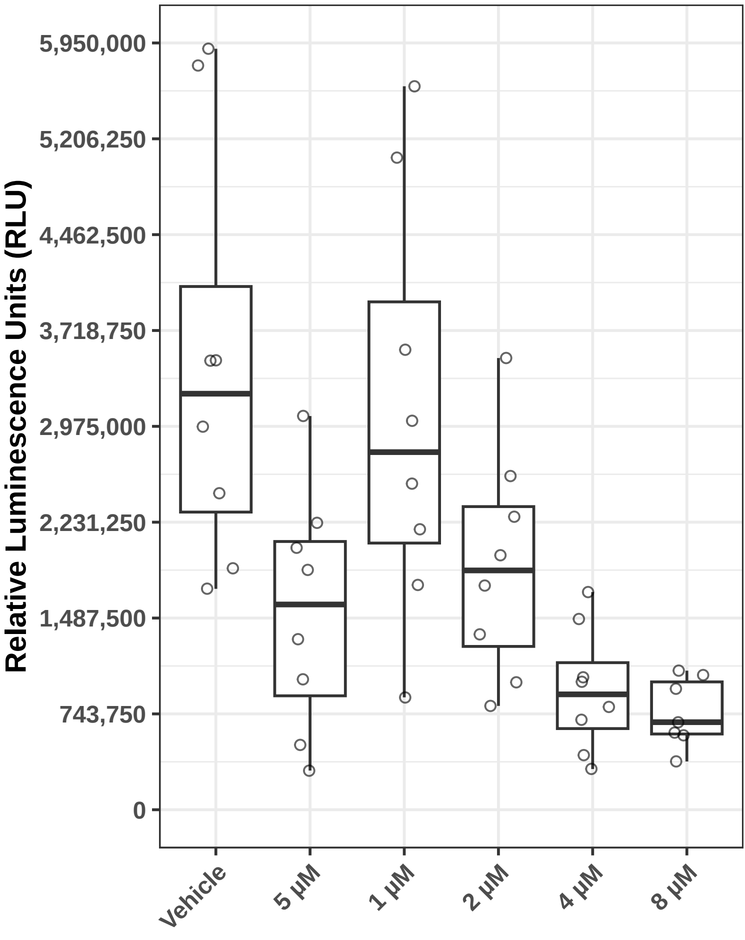

Relative Luminescence Units (RLU)
5,950,000
5,206,250
4,462,500
3,718,750
2,975,000
2,231,250
1,487,500
743,750
0
Vehicle
5 µM
1 µM
2 µM
4 µM
8 µM
